# An Excellent Example

**Published:** 1911-02-04

**Authors:** 


### An Excellent Example.

When the Liverpool School of Tropical Medicine lately lost its large-minded and generous founder, Sir Alfred Jones, the loss seemed at the moment to be one impossible to replace, notwithstanding the array of well-known and influential names which he had associated with himself as active members of the governing body. It would appear that in Mr. W. H. Lever, of Sunlight fame, a chairman has been found whose energy and other qualifications for this position render him no unworthy successor to Sir Alfred Jones himself. The Liverpool School is shortly sending out its twenty-seventh expedition for tropical research; and at a banquet held to entertain the members of it before they start for Gambia and Senegal, Mr. Lever put the case for these enterprises in a speech the report of which shows it to have been a most masterly exposition of his theme. The present expedition includes Professor J. L. Todd (Macdonald University, Canada); Dr. Walbach (Harvard University, U.S.A.); and Mr. E. D. Todd, a brother of the first-named, who is devoting himself to the collection of animals which assist in the spread of disease. Professor Todd was a member of the 1903 expedition to West Africa, ever memorable for the death of Dr. J. E. Dutton; and also of that of 1901. In proposing the toast of the expedition, Mr. Lever entered a strong plea for Governmental assistance towards the expense of these researches. It has been pointed out many times before that the work of rendering habitable the tropics is one the value of which to Europe, and especially to Britain, is beyond price; but the eloquent speech of the well-known soap-manufacturer may possibly, though it contains an oft-told tale, impress a Government of his own political complexion more than the weightiest pronouncements of professors and scientists who have no political influence. It is clear that Mr. Lever has assimilated thoroughly the main facts. As one who by reason of his business is largely interested in the export trade from West Africa and the tropics generally, Mr. Lever is well able to judge for himself the fearful waste of life and health entailed by the diseases which render the tropics so unhealthy for Europeans. The "wilful and preventable waste of money" caused by the invalidity of those whom Liverpool merchants send to West Africa to transact business there for them has impressed itself strongly on the speaker, who expressed in scathing terms his discontent with the torpor and inactivity of the Government in such matters. A pointed reference to the handling of the Panama problem by the United States authorities evoked loud applause.

Now there may seem to be nothing very remarkable in all this. An acute and far-sighted man of business has been convinced that tropical research as an economic investment is a good one; there is nothing very astonishing in that. But in point of fact there is much more in it. When Sir Alfred Jones died there was the chance that the driving force which he supplied to the Liverpool School might be cut off, too. For, indeed, it requires much more than mere conviction, much more also than mere pecuniary generosity, to make a successful chairman of such an institution. It needs a hard worker, and one with power to interest busy commercial men in a subject unfamiliar and entirely strange to them.

